# The influence of harsh parenting on adolescents' bullying: the moderating role of perceived class climate and gender

**DOI:** 10.3389/fpsyg.2026.1779353

**Published:** 2026-04-16

**Authors:** Zanxin Chen, Tongsheng Xi, Xiangrui Leng, Huiling Fan, Kun Cheng

**Affiliations:** 1 College of Education and Sports Sciences of Yangtze University, Jingzhou, Hubei, China; 2 College of Continuing Education of ZheJiang Gongshang University, Hangzhou, Zhejiang, China; 3 Experimental Primary School of Dadukou District of Chongqing, Chongqing, China; 4 School of Marxism of Yangtze University, Jingzhou, Hubei, China; 5 School of Foreign Studies of Yangtze University, Jingzhou, Hubei, China

**Keywords:** harsh parenting, adolescents' bullying, perceived class climate, gender, the moderating role

## Abstract

This study examined the moderating role of perceived class climate at school in the relationship between harsh parenting and adolescents' bullying and being bullied, as well as potential gender differences in this moderating mechanism. The sample included 408 adolescents aged 15–18 and their parents from a middle school in central China who completed self-report questionnaires. The results indicated that harsh parenting significantly predicted both bullying and being bullied among adolescents. Class climate played a significant moderating role: the associations between harsh parenting and bullying/being bullied were stronger under low class climate and weaker under high class climate, the moderating effect of gender was not significant.

## 1 Introduction

Bullying is generally defined as “intentional, repeated, and persistent negative behaviors of individuals or groups inflict toward victims in situations of power imbalance,” including verbal bullying, physical bullying, and relational bullying (Olweus, 1994). Bullying can cause serious psychological adaptation problems for both bullies and victims, affecting individual physical and mental development, people who frequently engage in bullying are prone to exhibit antisocial behavior, violations of discipline, laws and so on (Olweus, 1994). Individuals who are frequently bullied are prone to psychological distress such as anxiety, depression, and insecurity, and some may even exhibit non-suicidal self-injury (NSSI) and drug abuse (Behnken et al., 2010; Troop-Gordon, 2017). Mid-to-late adolescence is a critical developmental stage characterized by increased sensitivity to peer relationships and social status, during which bullying behaviors often become more salient (Bowes et al., 2015; Stahel et al., 2025). Therefore, identifying the meaning and influence factor of adolescents' bullying can help reduce it and promote the physical and mental health development of adolescents.

According to Social Learning Theory, individuals will learn different forms of aggressive behavior by observing and imitating their parents' behavior (Bandura and Walters, 1977). In recent years, the relationship between harsh parenting and adolescents' bullying has received some attention. Harsh parenting includes physical attacks (slapping, pushing), verbal attacks (yelling, insulting), and psychological attacks from parents toward adolescents (Wang et al., 2016; Simons et al., 1991). Research has found that harsh parenting is associated with adolescents' aggressive and destructive behavior, and is a family risk factor that triggers adolescent delinquency and substance abuse (Kim-Spoon et al., 2013a; Lansford et al., 2011). A study using parent-adolescent conflict measurement methods and multiple studies using harsh parenting scales have confirmed a significant positive correlation between harsh parenting and individual aggressive behavior (Miller-Perrin et al., 2009; Zhang, 2024; Ding et al., 2024). Conversely, the same harsh parenting environment may erode an adolescent's self-esteem and foster anxiety and withdrawal (Gershoff, 2002; Kim-Spoon et al., 2013b), potentially making them appear vulnerable and thus increasing their risk of being targeted by peers as a victim (Cook et al., 2010; Siegel and La Greca, 2009).

Grounded in ecological systems theory, adolescent development is shaped by the dynamic interplay of multiple environmental systems, among which family and school contexts are particularly influential. Family factors, such as parenting practices, play a critical role in shaping adolescents' behavioral outcomes. In the Chinese educational system, students are typically assigned to a fixed class group upon entering middle school and remain with the same classmates for most subjects across the school year, forming relatively stable peer relationships, teacher–student interactions, and class organization (Carman, 2012; Li, 2013). This stable class structure makes class climate an important contextual factor in adolescents' social development. Accordingly, the present study investigates how family and classroom environments jointly shape adolescents' bullying-related behaviors. Specifically, we examine whether harsh parenting predicts two distinct developmental outcomes—bullying and being bullied—and whether gender, perceived class climate moderates these associations.

### 1.1 Perceived class climate as a moderator

Adolescents' bullying is shaped by multiple contextual factors, including not only family influences such as harsh parenting but also the broader social and psychological environment at school (Jiang et al., 2012). Among school contexts, the classroom represents a central setting for students' daily activities and plays a crucial role in shaping their psychological development and behavioral adjustment (Zhang, 2023). As a key micro-system closely linked to adolescent development, the classroom environment influences not only students' acquisition of knowledge and skills but also the development of their personality, behavioral tendencies, and social functioning (Li et al., 2013). Supportive teacher-student relationships and high-quality emotional and instructional support from teachers contribute to stronger emotional bonds and foster a positive classroom atmosphere (Furrer et al., 2014). As an important component of the school social environment, class climate refers to students' perceptions of the quality of interpersonal relationships within the classroom, including the levels of cooperation, respect, and cohesion among classmates (Thornberg et al., 2024). A growing body of research suggests that positive class climate plays an important role in adolescents' social adjustment. Teacher support, guidance, and effective classroom management can improve peer relationships and weaken the negative association between aggressive behavior and peer acceptance among problem students (Guo and Zhang, 2004; Bai, 2018; Liu et al., 2020). From an ecological perspective, adolescents' behaviors are shaped not only by family environments but also by broader social contexts such as the classroom. A positive classroom climate characterized by supportive peer relationships and prosocial norms may buffer the negative effects of individual vulnerabilities. Empirical studies further indicate that class climate can moderate the associations between individual risk factors and social adjustment outcomes and may reduce the prevalence of bullying behaviors (Katulis and Kaniušonyte, 2023; Gazelle, 2006; Thornberg et al., 2025). Therefore, perceived class climate may play a role in mitigating the negative impact of harsh parenting on bullying.

### 1.2 Parent and adolescent gender differences in the moderating mechanism

Previous studies have shown that harsh parenting and adolescents' bullying may vary systematically based on parent and adolescent gender. Mothers showed more harsh discipline toward adolescents than fathers (Quach et al., 2015; McKee et al., 2007). Boys are more susceptible to harsh parenting, but girls who experience physical punishment from their parents are more likely to exhibit antisocial behavior (McKee et al., 2007; Tang, 2006; Deater-Deckard and Dodge, 1997). In addition, parental corporal punishment may lead to externalized problematic behaviors in boys (such as aggression and discipline), and internalized problematic behaviors such as anxiety and depression in girls (Gershoff, 2002). According to the Same-Sex Imitation Hypothesis, we can know that the influence of parenting styles is greatest in the combination of same-sex parents and adolescents (father and son, mother and daughter; Deater-Deckard and Dodge, 1997). Although previous studies have documented the parent and adolescent gender differences in harsh parenting and adolescents' bullying, little research has examined whether the moderating effects of perceived class climate on the relations between harsh parenting and adolescents' bullying differ by parent and adolescent gender.

Therefore, based on Ecological Systems Theory and the Same-Sex Imitation Hypothesis, this study predicts that: Parent and adolescent gender will significantly moderate the link between harsh parenting and bullying; The predictive power of harsh parenting on bullying, as well as the buffering effect of class climate.

## 2 Participants and methods

### 2.1 Participants

Ethical approval for this study was obtained from the Research Ethics Committee of Yangtze University. Participation was voluntary, and informed consent was obtained from both students and their parents or guardians before data collection.

Participants in this study were students from a middle school located in a province in central China. All questionnaires were completed by adolescents and their parents through self-report under the supervision of trained researchers. A total of 408 valid parent–adolescent dyads were included in the final. The majority of the participants were of Han ethnicity (98.75%), and all reported Mandarin Chinese as their native language. The adolescents had a mean age of 16.25 years (range = 15–18, SD = 0.89), and 56.75% were male. Regarding parental occupations, 82.5% of fathers and 70.54% of mothers were manual laborers (e.g., factory workers); approximately 12.16% of fathers and 8.66% of mothers were employed in professional or technical positions (e.g., teachers, physicians, or engineers); about 0.35% of fathers and 9.25% of mothers were unemployed. In terms of educational attainment, approximately 44.27% of fathers and 51.64% of mothers had completed junior high school, 34.27% of fathers and 30.15% of mothers had completed senior high school, and around 15.58% of fathers and 11.47% of mothers held a college degree or higher.

### 2.2 Research instruments

#### 2.2.1 Harsh parenting

Harsh parenting was measured using the Chinese version of the Harsh Parenting Scale (Kim-Spoon et al., 2013a; Chen and Li, 2009). The scale consists of four items (e.g., “telling the child to get out or even locking the child alone in a room”), rated on a five-point Likert scale ranging from 1 (never) to 5 (always). To reduce reporting bias and improve measurement stability, adolescents were also asked to evaluate their parents' parenting behaviors using parallel items with minor wording modifications (e.g., “When I do something wrong or make my parent angry, he/she loses his/her temper with me or even yells at me”). Four indicators were obtained: maternal harsh parenting (mother report), paternal harsh parenting (father report), maternal harsh parenting (adolescent report), and paternal harsh parenting (adolescent report). The Cronbach's alpha coefficients for these four measures were 0.715, 0.784, 0.754, and 0.760, respectively. The overall level of harsh parenting was calculated as the mean of these four measures, with higher scores indicating greater exposure to harsh parenting.

#### 2.2.2 Bullying

Bullying was assessed by the Chinese translated version of Bullying/Bullied Questionnaire (Zhang and Wu, 1999). Two subscales were selected to measure bullying/being bullied and the type of bullying/being bullied, each containing six items that assess three types of bullying behaviors: physical, verbal, and relational, totaling 12 items. Participants reported the frequency of bullying or victimization experiences over a recent period using a four-point scale (1 = “Never,” 2 = “Only once or twice,” 3 = “Three to five times,” 4 = “Six times or more”). The total scores for bullying and victimization were calculated by summing the respective item scores, with higher scores indicating higher levels of bullying or victimization. In the present study, the Cronbach's alpha coefficients for the bullying and victimization subscales were 0.748 and 0.768, respectively.

#### 2.2.3 Perceived class climate

Perceived class climate was measured by the Perceived Class Interpersonal Harmony Scale for students (Chen and Li, 2009). It is a 26-item scale with three dimensions of the perceived class climate, peer relationships (e.g., “Classmates support and encourage each other”), teacher-student relationships (e.g., “Students and teachers interact harmoniously”), class organization (e.g., “Students participate in class affairs and activities actively”), each item was rated on a five-point Likert-type scale ranging from 1 (never) to 5 (always). The mean score of each dimension represents its subscale score, and higher total scores indicate a more positive and harmonious interpersonal climate perceived by students (Li et al., 2015). The questionnaire demonstrated good reliability, with a Cronbach's alpha coefficient of 0.91 in the present study.

## 3 Results

### 3.1 Preliminary results and analysis

As all data in the present study were obtained from self-reported questionnaires, it was necessary to examine the potential influence of common method bias. An exploratory factor analysis using Harman's single-factor test was conducted on all items. The results indicated that the first factor accounted for 16.536% of the total variance, which is below the critical threshold of 40%. This suggests that common method bias was not a serious concern and could not explain most of the variance among the variables.

Given evidence from previous studies suggesting that gender may influence the present model, gender differences in harsh parenting, victimization, and bullying behaviors were examined (see Table 1). The results indicated no significant gender differences in harsh parenting (*t* = −1.053, *p* > 0.05), victimization (*t* = −1.006, *p* > 0.05), or bullying behavior (*t* = −0.521, *p* > 0.05). Therefore, no further group comparisons were conducted by gender.

**Table 1:** Gender differences in study variables.

Variable	Category	*M* ±*SD*	*t*	*p*
Harsh parenting	Boy	1.93 ± 0.83	−1.053	0.293
	Girl	2.02 ± 0.86		
Being bullied	Boy	1.99 ± 0.77	−1.006	0.315
	Girl	2.07 ± 0.83		
Bullying	Boy	2.01 ± 0.78	−0.521	0.603
	Girl	2.05 ± 0.79		

^*^p < 0.05, ^**^p < 0.01, ^***^p < 0.001.

### 3.2 Descriptive statistics, standard deviations, and correlation matrix of variables

Descriptive and correlational analyses were conducted using SPSS 26.0, and the results are presented in Table 2. Gender was not significantly correlated with harsh parenting, class climate, being bullied, or bullying. Harsh parenting was significantly and positively correlated with both bullying and being bullied, and negatively correlated with class climate. Being bullied was positively associated with bullying behavior and negatively associated with class climate. Bullying was significantly and negatively correlated with class climate.

**Table 2:** Descriptive statistics and correlations among study variables.

Research variable	*M*	*SD*	1	2	3	4	5
1 Gender^a^	0.56	0.50	–				
2 Harsh parenting	1.97	0.85	−0.052	–			
3 Bullying	2.03	0.79	−0.026	0.762^***^	–		
4 Being bullied	2.02	0.80	−0.050	0.747^***^	0.757^***^	–	
5 Class climate	3.78	0.64	−0.079	−0.198^***^	−0.220^***^	0.187^***^	–

^a^Gender was dummy-coded (0 = girl, 1 = boy).

^*^p < 0.05, ^**^p < 0.01, ^***^p < 0.001.

### 3.3 Moderating effects of class climate and gender

**Figure 1:**
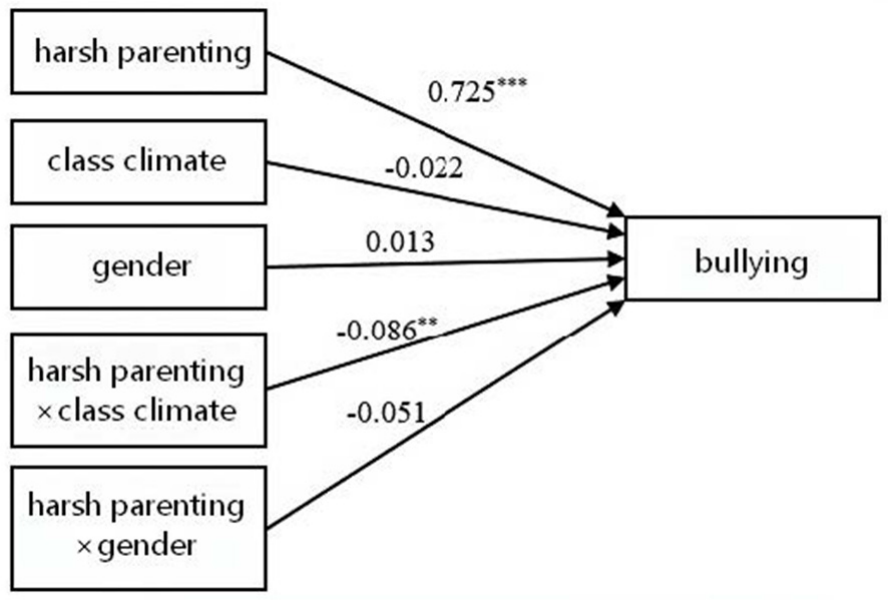
Model results of bullying. *p < 0.05, **p < 0.01, ***p < 0.001.

**Figure 2:**
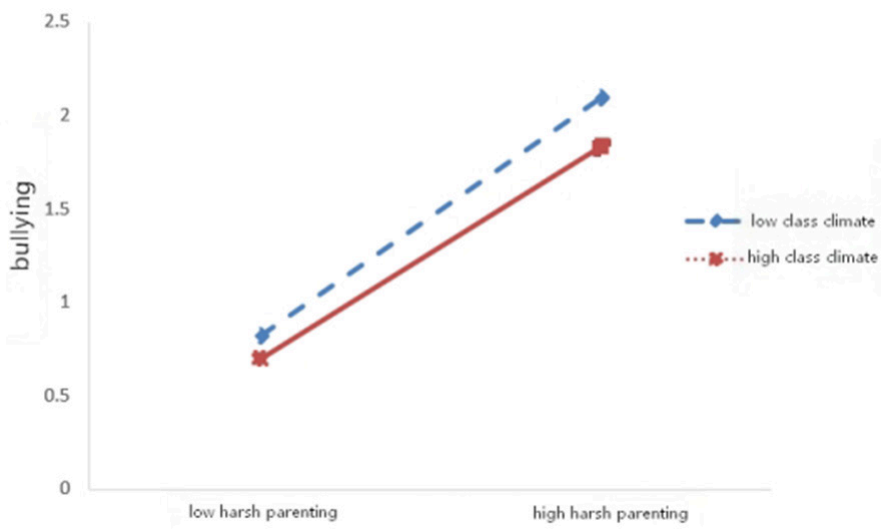
Moderating effect of classroom climate on bullying.

**Figure 3:**
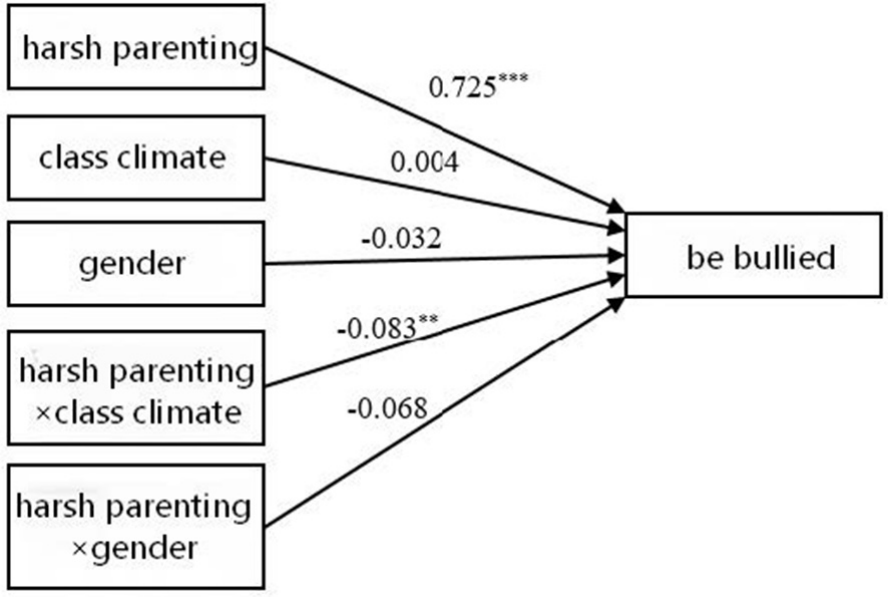
Model results of being bullied. *p < 0.05, **p < 0.01, ***p < 0.001.

**Figure 4:**
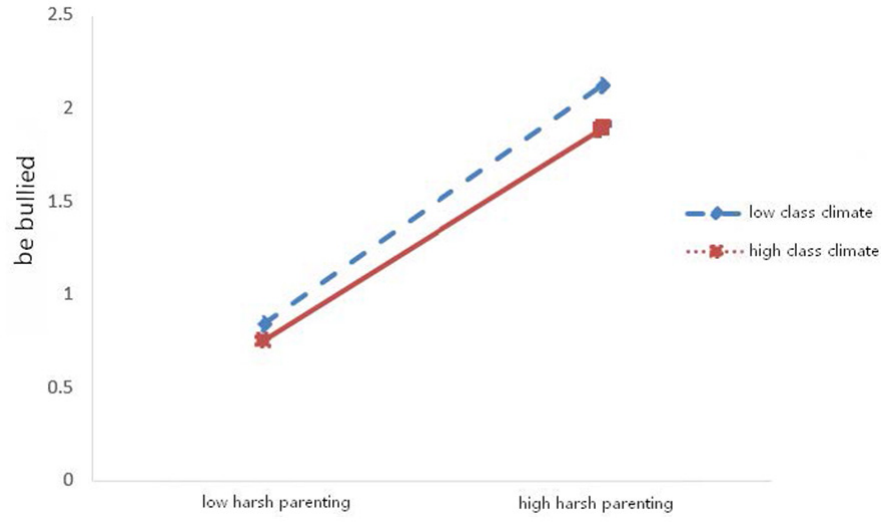
Moderating effect of classroom climate on being bullied.

First, the moderating effects of class climate and gender on the association between harsh parenting and adolescents' bullying behavior were examined (see Table 3 and Figure 1). The results indicated that harsh parenting significantly predicted adolescents' bullying behavior (β = 0.752, *p* < 0.001). The interaction term between harsh parenting and class climate was significant (β = −0.086, *p* < 0.01), whereas the interaction term between harsh parenting and gender was not significant (β = −0.051, *p* > 0.05). Simple slope analyses (see Figure 2) revealed that the predictive effect of harsh parenting on bullying behavior was stronger under low class climate (simple slope = 0.787, *t* = 17.129, *p* < 0.001) than under high class climate (simple slope = 0.615, *t* = 10.545, *p* < 0.001). Second, the moderating effects of class climate and gender on the relationship between harsh parenting and adolescents' victimization were tested (see Table 3 and Figure 3). The findings showed that harsh parenting significantly predicted. The interaction between harsh parenting and class climate was significant (β = −0.083, *p* < 0.01), while the interaction with gender was not significant (β = −0.068, *p* > 0.05; this finding should be interpreted with caution because the present study may not have had sufficient statistical power to detect potential gender differences in the moderating effect). Further simple slope analyses (see Figure 4) indicated that harsh parenting had a stronger predictive effect on victimization under low class climate (defined as −1 SD; simple slope = 0.766, *t* = 16.141, *p* < 0.001) than under high class climate (defined as +1 SD; simple slope = 0.601, *t* = 9.973, *p* < 0.001).

**Table 3:** Tests of the moderating effects of class climate and gender.

Regulated variable	Bullying			Be bullying		
	β	SE	*t*(*p*)	β	SE	*t*(*p*)
Harsh parenting	0.752	0.048	15.828 (*p* < 0.001)	0.752	0.049	15.310 (*p* < 0.001)
Class climate	−0.022	0.036	−0.613 (*p >* 0.05)	0.004	0.037	0.113 (*p >* 0.05)
Gender	0.013	0.065	0.196 (*p >* 0.05)	−0.032	0.066	−0.485 (*p >* 0.05)
Harsh parenting × class climate	−0.086	0.027	−3.218 (*p* < 0.01)	−0.083	0.028	−2.992 (*p* < 0.01)
Harsh parenting × gender	−0.051	0.065	−0.788 (*p >* 0.05)	−0.068	0.067	−1.017 (*p >* 0.05)
	*R*^2^ = 0.596, *F*_(5, 402)_ = 1,118.66, *p* < 0.001			*R*^2^ = 0.569, *F*_(5, 402)_ = 106.222, *p* < 0.001		

SE, Standard Error.

^*^p < 0.05, ^**^p < 0.01, ^***^p < 0.001.

## 4 Discussion

The present study examined the associations between harsh parenting and adolescents' bullying and being bullied, as well as the moderating roles of class climate and gender. Based on a sample of 408 Chinese junior high school students and their parents, the findings revealed that harsh parenting significantly predicted both bullying and being bullied among adolescents. Class climate played a significant moderating role: the associations between harsh parenting and bullying/being bullied were stronger under low class climate and weaker under high class climate, the moderating effect of gender was not significant. These findings contribute to understanding how family and school environments jointly influence adolescents' social behaviors from the perspective of ecological systems theory.

### 4.1 The impact of harsh parenting on adolescent bullying

Consistent with previous findings, harsh parenting was positively associated with both adolescents' bullying and being bullied (Kim-Spoon et al., 2013b; Lansford, 2011; Wang and Liu, 2018). According to social learning theory, children internalize their parents' aggressive behavioral patterns within the family and replicate them in peer interactions (Bandura and Walters, 1977). Frequent scolding, threats, or corporal punishment convey to children that aggression is an acceptable means of resolving conflicts, thereby increasing the likelihood of engaging in bullying behaviors at school. Meanwhile, adolescents raised in harsh parenting environments may develop anxiety and withdrawal tendencies, leading them to exhibit submissiveness or low self-esteem in peer interactions, which in turn makes them more vulnerable to be bullied (Gershoff, 2002). This dual risk of “bullying and being bullied” suggests that harsh parenting not only exacerbates externalizing problems but also triggers internalizing distress, exerting persistent negative effects on adolescents' social adjustment.

### 4.2 The moderating role of perceived class climate

The present study found that class climate played a significant moderating role in the relationship between family factors and bullying, consistent with the core assumption of ecological systems theory—that children's development is shaped by the interactions among multiple environmental systems (Bronfenbrenner, 2013). The findings indicated that a positive class climate can buffer the detrimental effects of harsh parenting on adolescents' bullying and being bullied. Conversely, when the class climate is poor and lacks support and cohesion, the negative influence of harsh parenting tends to be amplified. In other words, a harmonious and supportive class environment provides adolescents with emotional support and prosocial norms, thereby mitigating the spillover effects of family-related risks. This finding aligns with prior research showing that supportive teacher–student and peer relationships help reduce aggressive behaviors and promote social adjustment (Li et al., 2013; Thornberg et al., 2023). Furthermore, these findings may extend the understanding of the healthy context paradox, which suggests that individuals experiencing adversity may sometimes experience heightened sensitivity to social contexts. In the present study, adolescents exposed to harsh parenting showed different levels of bullying behavior depending on the perceived classroom climate. This indicates that the broader social environment may shape how family-based risk factors are expressed in peer interactions. Future research should further examine the conditions under which a positive class climate might paradoxically become a risk factor (Salmivalli, 2010).

### 4.3 The non-significant moderating effect of gender

Contrary to the initial hypothesis, gender did not significantly moderate the associations between harsh parenting and adolescents' bullying or victimization behaviors. One possible explanation is that gender socialization in contemporary Chinese society is shifting toward greater equality, leading to a gradual reduction in behavioral differences between males and females. Large-scale empirical studies have shown that younger generations in China increasingly endorse egalitarian gender beliefs, and the expansion of educational opportunities has significantly contributed to the development of gender-equal attitudes (Du et al., 2021; Zhang and Liu, 2022). As societal emphasis on gender equality and emotional expression increases, the traditional behavioral expectations of “male externalization and female internalization” have become less evident among contemporary adolescents. Moreover, recent empirical evidence suggests that gender differences in emotional and behavioral adaptation are context-dependent and are diminishing over time. For example, Gong et al. (2023) found partial overlap in the developmental trajectories of internalizing and externalizing problems between boys and girls from middle childhood to adolescence. Similarly, a large-scale study of Shanghai high school students by Zhang et al. (2024) reported no significant gender differences in the overall network structure of psychological symptoms. These findings suggest that although males and females may differ in specific behavioral expressions, the underlying mechanisms through which harsh parenting affects adolescents—such as emotional dysregulation and modeling of aggression—may operate similarly across genders. Therefore, the non-significant moderating effect of gender observed in this study may reflect an ongoing convergence in socialization and emotional regulation patterns among Chinese adolescents. Additionally, the study may not have been adequately powered to detect gender differences in the moderating role of class climate. Future studies with larger and more diverse samples, employing longitudinal and multi-informant designs, are needed to further examine potential gender differences in socialization processes and to distinguish between physical and relational bullying, thereby identifying more nuanced gender-specific pathways within evolving sociocultural contexts.

### 4.4 Limitations and future directions

This study has several limitations due to various factors such as survey conditions. First, all data were obtained through self-reports. Considering that experiences of harsh parenting or peer bullying are sensitive and socially undesirable topics, not all adolescents may have been willing to report them truthfully. Some participants might have concealed the facts or downplayed the severity of their experiences, potentially leading to discrepancies between the data and the actual situation. Second, this study adopted a cross-sectional design, and therefore the findings should be interpreted with caution. Longitudinal studies are needed to further validate the results and enhance the reliability and scientific rigor of the conclusions. Third, although gender was included as a grouping variable, the study was not powered to test whether the moderating effect of class climate differs between boys and girls. Prior research suggests that boys tend to respond more strongly to classroom disorder and conflict, whereas girls are more sensitive to relational and emotional aspects of the classroom environment. Future research should therefore examine gender-specific moderation models to clarify whether class climate provides different protective or risk-amplifying effects for boys and girls. Fourth, the participants in this study shared a relatively homogeneous sociocultural background, which may limit the applicability and generalizability of the findings to multicultural contexts. Hence, further research is warranted to examine the broader generalizability of these results across diverse cultural settings. Finally, all participants were recruited from the same school. This sampling strategy may limit the variability of contextual factors and restrict the generalizability of the findings. Although students reported different perceptions of class climate, they were still embedded within the same broader school environment, which may have reduced variability in classroom contexts. Future studies should include participants from multiple schools to capture greater diversity in classroom environments.

### 4.5 Conclusion

In summary, this study elucidates the significant impact of harsh parenting on adolescent bullying and victimization behaviors and reveals the critical contextual moderating role of class climate. Our findings enrich ecological systems theory by demonstrating a synergistic interaction between the family microsystem and the class microsystem, whereby positive teacher–student and peer relationships can buffer the adverse effects of negative parenting practices. Practically, these results advocate for a “dual-system synergy” intervention approach that combines parent training programs aimed at reducing harsh discipline and enhancing parental emotional responsiveness with school-wide efforts to foster an inclusive, supportive, and cooperative class climate, thereby strengthening students' sense of belonging and security. Although gender differences were not significant, this study underscores the necessity of coordinated efforts between family and school to effectively prevent bullying and promote adolescents' psychological wellbeing and social adjustment.

## Data Availability

The original contributions presented in the study are included in the article/supplementary material, further inquiries can be directed to the corresponding author.
